# *Mest/Peg1* Is Essential for the Development and Maintenance of a SNc Neuronal Subset

**DOI:** 10.3389/fnmol.2016.00166

**Published:** 2017-01-13

**Authors:** Simone Mesman, Johannes A. van Hooft, Marten P. Smidt

**Affiliations:** Swammerdam Institute for Life Sciences, FNWI University of AmsterdamAmsterdam, Netherlands

**Keywords:** midbrain, dopamine, neuronal development, degeneration, Parkinson’s disease

## Abstract

Mesodiencephalic dopaminergic (mdDA) neurons originate at the floor plate and floor plate-basal plate boundary of the midbrain ventricular zone. During development mdDA neurons are specified by a unique set of transcription factors and signaling cascades, to form the different molecular subsets of the mdDA neuronal population. In a time series micro-array study performed previously, mesoderm specific transcript (*Mest*) was found to be one of the most upregulated genes during early mdDA neuronal development. Here, we show that *Mest* transcript is expressed in the midbrain throughout development and becomes restricted to the substantia nigra (SNc) at late stages. In *Mest* KO animals mdDA neurons are progressively lost in the adult, mostly affecting the SNc, reflected by a 50% decrease of TH protein and DA release in the striatum and a reduction of climbing behavior. Analysis of *Lrp6* KO embryos suggest a subtle opposite phenotype to the *Mest* KO, hinting toward the possibility that specific loss of mdDA neurons in *Mest* ablated animals could be due to affected WNT-signaling. Interestingly, the mdDA neuronal region affected by the loss of *Mest* remains relatively unaffected in *Pitx3* mutants, suggesting that both genes are essential for the development and/or maintenance of different mdDA neuronal subsets within the SNc. Overall, the neuroanatomical and phenotypical consequences detected upon the loss of *Mest*, resemble the loss of SNc neurons and loss of movement control as seen in Parkinson’s Disease (PD), suggesting that the *Mest* mouse model may be used as a model-system for PD.

## Introduction

The mesodiencephalic dopaminergic (mdDA) group of neurons is involved in motivation, reward, movement, and addiction. It consists of different molecular subsets that are specified during embryogenesis by a unique set of transcription factors and signaling cascades (Smits et al., 2006, 2013; Di Salvio et al., 2010; Jacobs et al., 2011; Hoekstra et al., 2013; Veenvliet et al., 2013). The presence of different molecular subsets in the mdDA neuronal population is nicely illustrated by the selective loss of mdDA neurons of the substantia nigra (SNc) during Parkinson’s Disease (PD). To gain more insight in developmentally regulated genes that are involved in mdDA differentiation and subset specification, we previously conducted a genome-wide expression study on the ventral midbrain at different embryonic stages (Chakrabarty et al., 2012). From that data it was clear that many genes peak specifically during the early specification phase of dopaminergic neurons. Some of these genes remain expressed and others show a gradual decline in expression during development. Since mdDA neurons are born from embryonic day (E)10.5 onward (Bayer et al., 1995), genes that are highly expressed at early stages of development could play a role at the (early) specification and differentiation of mdDA neurons. From this data-set we identified mesoderm specific transcript (*Mest*) as one of the most upregulated genes during early development.

*Mest*, also known as paternally expressed gene 1 (*Peg1*), is one of the first identified epigenetically regulated genes (Kaneko-Ishino et al., 1995). The maternal allele is silenced via extensive methylation of the promoter region, which is established after fertilization (Lefebvre et al., 1997, 1998). Methylation of the maternal allele is normally permanent, although it has been shown that heterozygous mice that do not have a functional paternal allele can show expression of *Mest* after E13.5, indicating that imprinting of the maternal allele can be reversed under certain conditions (Imamura et al., 2005; Ineson et al., 2012). *Mest* knock-out (KO) mice are viable and, apart from growth retardation, have not been reporter to show obvious phenotypic features (Lefebvre et al., 1998). Sporadically, female KO mice show a loss of maternal care, resulting in the need of fostering paradigms (Lefebvre et al., 1998). The fact that not all *Mest* deficient mice exhibit this phenotype, suggests that the function of *Mest* is influenced by environmental and genetic background variability (Lefebvre et al., 1998).

*Mest* is a member of the divergent α-β hydrolase protein family, characterized by the presence of 8 β-sheets connected by α-helices in the core of the protein (Ollis et al., 1992; Tilghman, 1999). Most members of this family are regulators of lipid metabolism, although some have directly been implicated in signaling pathways that are involved in the enzymatic synthesis and degradation of key signaling lipids (Lord et al., 2013). The exact function of *Mest* in mice, and especially in the brain, is unknown. During adipocytogenesis *Mest* has been found to inhibit LRP6 glycosylation and is thereby able to regulate the balance of non-canonical and canonical WNT-signaling (Jung et al., 2011). This type of signaling is also known to be relevant during brain development, suggesting that *Mest* may serve a similar function during development of the central nervous system. Both canonical and non-canonical WNT-signaling have been reported to be important in the development and specification of mdDA neurons (Castelo-Branco et al., 2004; Schulte et al., 2005; Cajánek et al., 2009; Mesman et al., 2014). If MEST similarly regulates the maturation of LRP6 during mdDA neuronal development, it could play an important role in the onset of mdDA neuronal specification and differentiation.

Here, we show that *Mest* is expressed in the developing mouse midbrain and overlaps with mdDA neurons throughout development and in the adult. At late developmental stages and in the adult midbrain, the expression of *Mest* becomes restricted to (part of) the SNc. *Mest* KO mice show a progressive loss of mdDA neurons between 3- and 8-months, which is most severe in the SNc. The loss of mdDA neurons in the SNc of the adult *Mest* KO animals is reflected by a 50% loss of TH-protein level and a 50% decrease in DA release in the striatum. The loss of mdDA neurons in the midbrain may be influenced by control of WNT-signaling via regulation of LRP6 maturation by MEST, as *Lrp6* KO embryos show a partial opposite phenotype to the *Mest* KO. Interestingly, we detected that the regions mostly affected by the loss of *Mest* are relatively intact in the *Pitx3* KO, suggesting that *Pitx3* and *Mest* regulate the development and/or maintenance of two different parts of the SNc. We conclude that *Mest* is a novel factor in the development and maintenance of (a subset of) neurons of the SNc. The progressive loss of mdDA neurons in the SNc of the *Mest* KO and consequently the decrease in DA release in the striatum and the decrease in climbing activity resembles mdDA neuronal loss and the phenotypical consequences as seen in PD.

## Materials and Methods

### Ethics Statement

All animal studies were performed in accordance with local animal welfare regulations, as this project has been approved by the animal experimental committee (Dier ethische commissie, Universiteit van Amsterdam, DEC-UvA), and international guidelines.

### Animals

The transgenic *Mest*^tm1Lef^ strain was a generous gift of Prof. Dr. Azim Surani from the Wellcome Trust/Cancer Research UK Gurdon Institute of the University of Cambridge (Lefebvre et al., 1998). *Mest*^tm1Lef^ mouse-line was back-crossed with the C57BL/6 line and KO and wild type (WT) embryos and adults were generated by crossing heterozygous *Mest* mutant mice. As no differences were expected between males and females, both genders were used in the experiments performed. WT embryos and adults to study normal gene expression patterns were generated by crossing C57BL/6 mice. Pregnant [embryonic day 0.5 (E0.5) was defined as the morning of plug formation] and adult mice were sacrificed by cervical dislocation. Embryos and brains were collected in 1x PBS and immediately frozen on dry-ice, or fixed by immersion for 3–12 h in 4% paraformaldehyde (PFA) at 4°C. After PFA incubation, samples were washed in 1x PBS and cryoprotected O/N at 4°C in 30% sucrose. Embryos and brains were frozen on dry-ice and stored at -80°C. Cryosections were sliced at 16 μm, mounted on Superfrost Plus slides (Thermo Fisher Scientific), air-dried, and stored at -80°C until further use.

E17.5 *Lrp6* KO embryonic tissues were a kind gift of Prof. Dr. E. Arenas from the Karolinska Institute in Stockholm, Sweden.

### Genotyping

Genotyping of the *Mest*^tm1Lef^ strain was performed as described (Lefebvre et al., 1997). In short: PCR was performed in two reactions. The first reaction contains 50 ng of genomic DNA together with a common FP 5′-CTGGCTGCGTACCTGCACATC-3′ and RP1 5′-GCTTCCGACCACACCGACAG-3′ binding at respectively exon 2 and exon 3 of the *Mest* gene, resulting in a WT product of 1.4 kb. The second reaction contains the common FP and RP3 5′-AGACCGCGAAGAGTTTGTCCTC-3′ binding at respectively exon 2 of the WT gene and the pIFS sequence upstream of the IRES, resulting in a 1.15 kb-product of the mutant allele.

### Immunohistochemistry

Fluorescence immunohistochemistry was carried out as described previously (Kolk et al., 2009; Fenstermaker et al., 2010). Cryosections were blocked with 4% heat inactivated fetal calf serum (HIFCS) or 5% normal donkey serum (for sheep primary antibodies) in 1x THZT and incubated O/N with a primary antibody [Rb-TH (Pelfreeze 1:1000), Sh-TH (Millipore AB1542, 1:1000)]. The next day sections were incubated with a secondary Alexafluor antibody (anti-rabbit, anti-sheep) diluted 1:1000 in 1x TBS for 2 h at RT (20°C ± 1°C). After extensive washing in 1x PBS, slides were embedded in Fluorsave (Calbiogen) and analyzed with the use of a fluorescent microscope (Leica).

Quantification of TH-expressing cells in the adult and E17.5 midbrain was performed in ImageJ as follows. TH-expressing cells of the SNc, ventral tegmental area (VTA), and both, were counted in 8–12 (adult) (WT *n* = 3, KO *n* = 3) and 6 (E17.5) (WT *n* = 2; KO *n* = 2) (matching) coronal sections. Cells were counted as TH^+^ cells when TH staining co-localized with a nuclear DAPI-staining. The SNc and VTA were determined based on anatomical landmarks. The SNc was clearly determined in sections rostral from the fasciculus retroflexus, whereas the mdDA area was divided in the VTA and SNc in sections containing, and caudal of, the fasciculus retroflexus. The distinction between the SNc and VTA was made based on the tracts of the medial lemniscus, positioned in between the SNc and VTA. Statistical analysis was performed via a one-tailed student’s *t*-test.

Intensity of TH-levels in the striatum of *Mest* WT and KO animals was measured with the use of ImageJ, as described previously (McCloy et al., 2014). Intensity was measured in 6 matching coronal slices in dorsal and ventral parts of the striatum (WT *n* = 3, KO *n* = 4). After retraction of the background intensity (normalization) we performed statistical analysis via a one-tailed student’s *t*-test.

### *In situ* Hybridization and Combined TH-DAB IHC

*In situ* hybridization with digoxigenin (DIG)-labeled probes was performed as described previously (Smidt et al., 2004). Fresh frozen sections were fixed in 4% PFA for 30 min and acetylated with 0.25% acetic anhydride in 0.1 M triethanolamine for 10 min. Probe hybridization was carried out at 68°C O/N with a probe concentration of 0.4 ng/μl in a hybridization solution containing 50% deionized formamide, 5x SSC, 5x Denhardt’s solution, 250 μg/ml tRNA Baker’s yeast, and 500 μg/ml sonificated salmon sperm DNA. The following day slides were washed in 0.2x SSC for 2 h at 68°C followed by blocking with 10% HIFCS in buffer 1 (100 mM TricHCl, pH = 7.4 and 150 mM NaCl) for 1 h at RT. DIG-labeled probes were detected by incubating with alkalin-phosphatase-labeled anti-DIG antibody (Roche, Mannheim), using NBT/BCIP as a substrate.

DIG *in situ* hybridization was performed with the following probes: 491 bp *Th* fragment bp 252–743 of mouse cDNA, 359 bp *Dat* fragment bp 762–1127 of rat cDNA, 648 bp *Mest* fragment bp 648–1298 of mouse cDNA, 993 bp *Lrp6* fragment bp 1015–2008 of mouse cDNA. If *in situ* hybridization was not followed by TH-DAB IHC, slides were washed 2x5 min in T_10_E_5_, dehydrated with ethanol, and embedded in Entellan.

After *Mest* and *Lrp6* DIG *in situ* hybridization, sections were immunostained for TH. Slides were incubated in 0.3% H_2_O_2_ in Tris-buffered saline (TBS) for 30 min at RT. Thereafter, blocking was performed with 4% HIFCS in TBS. Slides were incubated O/N with primary antibody Rb-TH (Pelfreeze, 1:1000) in TBS. The following day slides were incubated for 1 h with goat-anti-rabbit biotinylated secondary antibody (Vector, 1:1000) in TBS, followed by incubation with avidine-biotin-peroxidase reagents (ABC elite kit, Vector Laboratories 1:1000) in TBS. The slides were stained with DAB (3,3′-diamino-benzidine) for a maximum of 10 min, dehydrated with ethanol and embedded with Entellan.

### Nissl Staining

Nissl staining was performed as described previously (Veenvliet et al., 2013). Fresh frozen sections of 3- and 8-month old *Mest* KO and WT littermates were used.

### Climbing Test

The climbing test was performed as described previously (Costall et al., 1978) Smidt et al., 2004 during the active phase of the mice. Three-months old mice were placed in climbing cages and acclimatized for 30 min. Statistical analysis was performed via a two-tailed student’s *t*-test.

### Fast Cyclic Voltammetry

Mice were sacrificed by cervical dislocation and 250 μm-thick coronal brain slices were prepared on a vibroslicer (Leica VT1000S) using ice-cold modified artificial cerebrospinal fluid (ACSF) containing 120 mM choline chloride, 3.5 mM KCl, 0.5 mM CaCl_2_, 6 mM MgSO_4_, 1.25 mM NaH_2_PO_4_, 25 mM NaHCO_3_, and 25 mM glucose, continuously bubbled with 95% O_2_-5% CO_2_ (pH = 7.4). Slices containing the striatum were kept submerged in a recording chamber at RT and superfused with recording ACSF containing 120 mM NaCl, 3.5 mM KCl, 2.5 mM CaCl_2_, 1.3 mM MgSO_4_, 1.25 mM NaH_2_PO_4_, 25 mM NaHCO_3_, and 25 mM glucose, continuously bubbled with 95%O_2_-5%CO_2_.

Carbon fibers for recording were made in house. A ∼9 μm thick carbon fiber (generous gift of Dr. Ingo Willuhn, NIN, Amsterdam, The Netherlands) was aspirated in a boroscilicate glass capillary using vacuum suction. The capillary was subsequently pulled on a horizontal electrode puller (P-97, Sutter Instruments, Novato, USA). The exposed carbon–fiber was trimmed to approximately 0.5 mm and the fiber was glued and coated with transparent nail polish, leaving ∼100 μm of the tip exposed. The carbon-fiber electrode was backfilled with 1M KCl and connected to the headstage of an EPC9 patch clamp amplifier controlled by PULSE software (HEKA Electronics, Lambrecht, Germany). The carbon-fiber was placed in either the dorsal of ventral striatum and lowered into the slice (∼50 μm below the surface) at ∼100 μm distance from a bipolar stimulation electrode. DA release was evoked by electrical stimulation (10–500 μA, 1 ms) and recorded by ramping the potential of the carbon-fiber from -500 mV to 1 V and back to -500 mV at 300V/s at 10 Hz vs. an Ag/AgCl reference electrode. Signals were filtered at 3 kHz and samples at 10 kHz. Voltammograms obtained before stimulation were off-line subtracted from those obtained after stimulation using Igor Pro (Wavemetrics, Tigard, OR, USA). Carbon-fibers were calibrated in ACSF containing 1–10 μM DA before and after each experiment. Statistical analysis was performed via a two-tailed student’s *t*-test.

## Results

### *Mest* Is Expressed in the Midbrain during Development and Remains Expressed in the Adult SNc

**FIGURE 1:**
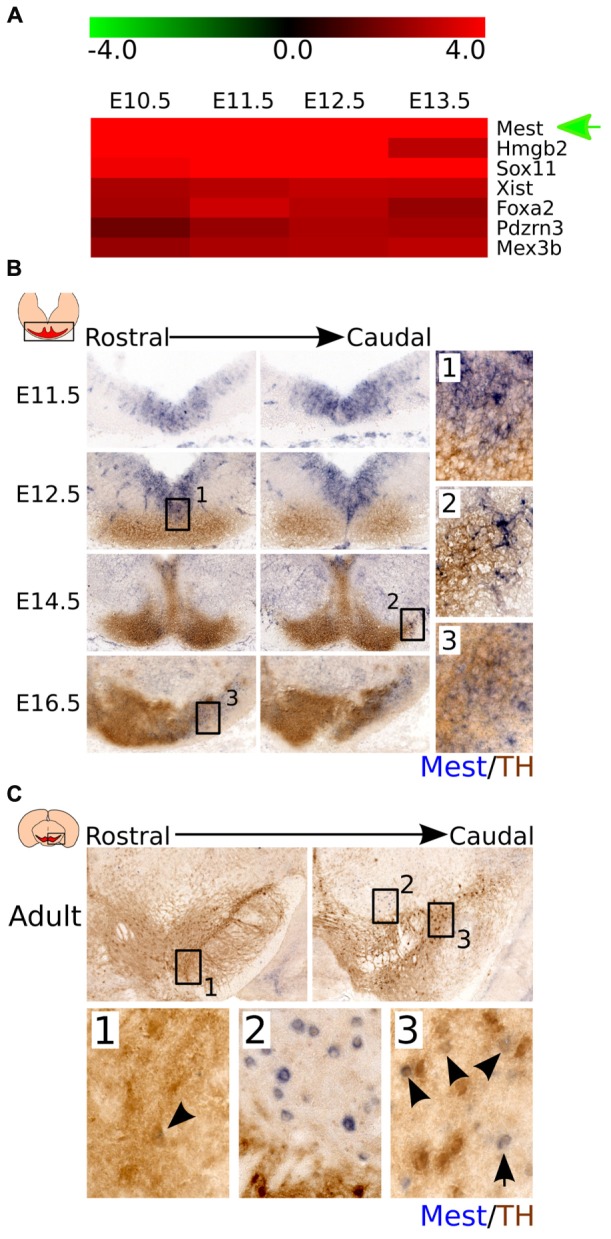
***Mest* is highly expressed during development and shows a specific expression in the midbrain area.** **(A)** *Mest* is one of the most upregulated genes in developmental stages compared to adult mdDA neurons. **(B)** Coronal view of *Mest* expression throughout development. *Mest* (blue) is specifically expressed in the FP and BP of the developing midbrain throughout development. At E12.5 some overlap can be detected with the rostral TH-expressing population (brown) **(1)**, whereas at later stages *Mest* expression becomes specified to the future SNc (**2** and **3**). **(C)** Coronal view of *Mest* expression in the adult mdDA neuronal population. *Mest* (blue) expression can be detected in some TH-expressing neurons (brown) of the SNc (**1** and **3**), although *Mest* transcript can also be detected in cells surrounding the TH^+^ neurons of the SNc **(2)**.

To investigate which genes could be important during mdDA neuronal birth and later during neuronal differentiation, we performed meta-analysis on the time-line genome-wide expression study performed earlier in our lab (Chakrabarty et al., 2012). We found that, amongst other factors, *Mest* is highly expressed during the early phase of mdDA neuronal development (**Figure 1A**). Its high expression early during development suggests that it could be an important factor in the onset of mdDA neuronal birth and initial differentiation.

**FIGURE 2:**
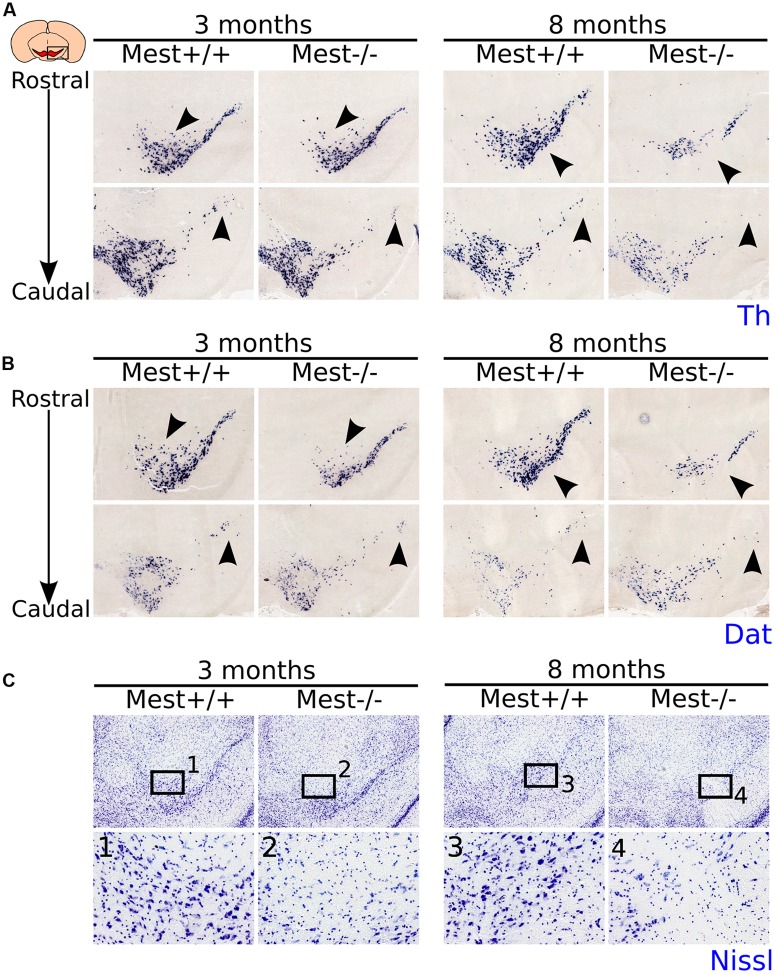
**MdDA neurons are lost in the midbrain of adult *Mest* KO animals.** **(A)** *Th*-expression (blue) is affected in the SNc of 3- and 8-months old adult KO mice. The loss of *Th* expression seems to be progressive as more *Th*^+^ cells are lost in the 8-months old midbrain (black arrowheads). **(B)** *Dat*-expression (blue) is similarly affected in the SNc of 3- and 8-months old adult KO mice as *Th-*expression (adjacent sections) (black arrowheads). **(C)** Nissl-staining on adjacent sections at 3- and 8-months old *Mest* KO and WT controls shows that the loss of *Th* and *Dat* is accompanied by a decreased DA cell density in the SNc (black arrowheads), indicating that *Mest* depletion results in a mdDA cell loss and not in a specific loss of mdDA markers.

**FIGURE 3:**
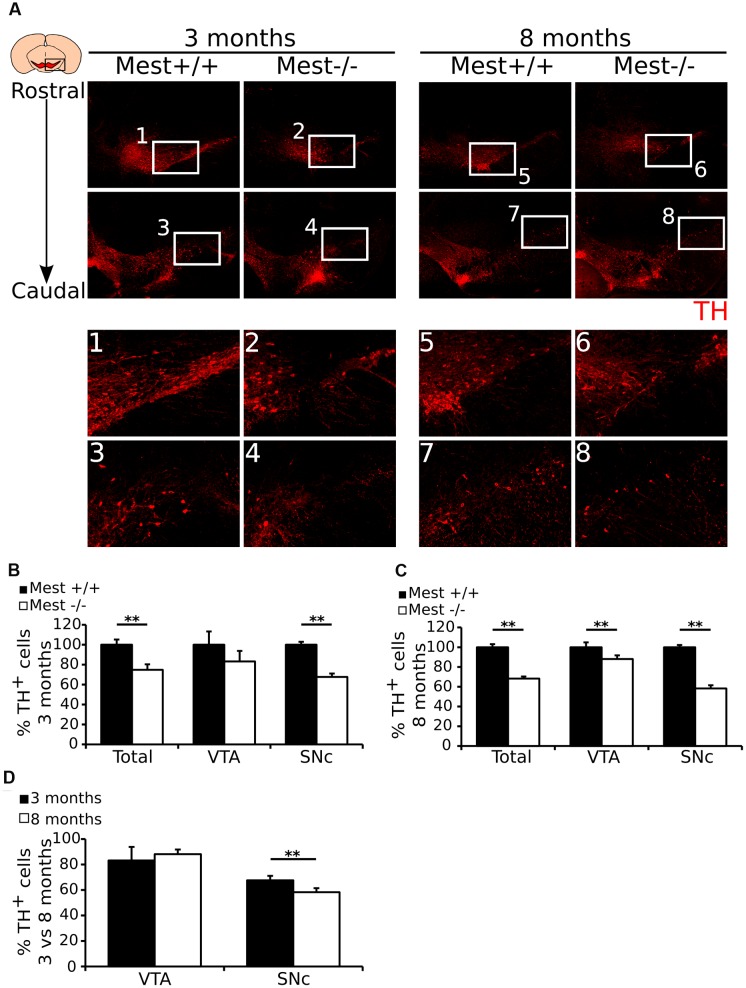
***Mest* ablated animals show a specific and progressive loss of mdDA neurons in the SNc.** **(A)** Protein expression of TH (red) is lost in the SNc of the adult mdDA system of 3- **(1–4)** and 8-months **(5–8)** old *Mest* KO mice, similarly to the loss of *Th* transcript. **(B)** Quantification of TH protein in the midbrain of 3-months old adult *Mest* KO (*n* = 3, white bars) and *Mest* WT controls (*n* = 3, black bars) shows that in the 3-months old midbrain the total amount of TH^+^ neurons is significantly lower in the KO than in the WT (∼25% loss, ^∗∗^*p* < 0.01), which is reflected in the TH-expressing population in the SNc (∼32% loss, ^∗∗^*p* < 0.01), but not of the VTA. WT was set at 100%. **(C)** Quantification of TH protein in the midbrain of 8-months old adult *Mest* KO (*n* = 3, white bars) and *Mest* WT controls (*n* = 3, black bars) shows that in the 8-months old midbrain the total amount of TH^+^ neurons is significantly lower in the KO than in the WT (∼32% loss, ^∗∗^*p* < 0.01), which is reflected in the TH-expressing population in the SNc (∼42% loss, ^∗∗^*p* < 0.01), and also in the VTA (∼12% loss, ^∗∗^*p* = 0.01). WT was set at 100%. **(D)** Comparison of the loss of cells in the mdDA neuronal population between 3- and 8-months old animals shows that the loss of cells in the VTA of 8-month old animals is not significantly altered, whereas the loss of cells in the SNc of 8-months old animals shows a progressive loss (∼32% vs. ∼42% loss, ^∗∗^*p* = 0.01). WT was set at 100%.

To validate the data from the time-line genome-wide expression study and define the spatial distribution of *Mest* in the midbrain area, we analyzed *Mest* transcript expression by means of *in situ* hybridization during differentiation (**Figure 1B**) and in the adult stage (**Figure 1C**). *Mest* transcript is detected from E11.5 onward and remains expressed in the adult midbrain. At E11.5 *Mest* is mainly expressed in the floor plate (FP) and basal plate (BP) of the ventricular zone (VZ) where it remains expressed at least until E14.5. At E12.5 *Mest* overlaps with a small part of the medial TH-expressing neuronal population in rostral areas (**Figure 1B-1**). Whereas at E14.5 *Mest* expression is decreased in the VZ and displays a broad expression, partially overlapping at lateral-caudal areas with TH expression (**Figure 1B-2**). Later during development, at E16.5, *Mest* is most prominently expressed in the future SNc region (**Figure 1B-3**). In the adult mdDA area *Mest* transcript can be detected in some TH-expressing cells of the SNc (**Figure 1C-1** and **3** black arrowheads) and in cells surrounding nigral mdDA neurons (**Figure 1C-2**). Together, the spatial-temporal expression data confirmed the initial time-line genome-wide expression profile. At early developmental stages *Mest* is strongly expressed in the midbrain and shows a relatively broad expression pattern. At late developmental stages *Mest* becomes more restricted to lateral parts of the TH-expressing population and is eventually present in neurons located in the SNc (adult stage).

### Ablation of *Mest* Induces a Progressive Loss of DA Neurons in the SNc

The presence of *Mest* in the VZ at early developmental stages and specifically in the SNc at late developmental stages, suggests that it may be involved in mdDA neuronal development and adult function of mdDA neurons located in the SNc. To investigate the role of *Mest* in the neurochemical and neuroanatomical integrity of the mdDA system, we examined the presence of the mdDA neuronal marker TH in *Mest* KO animals at E14.5 and E17.5, and both *Th* and *Dat* in the adult stage.

**FIGURE 4:**
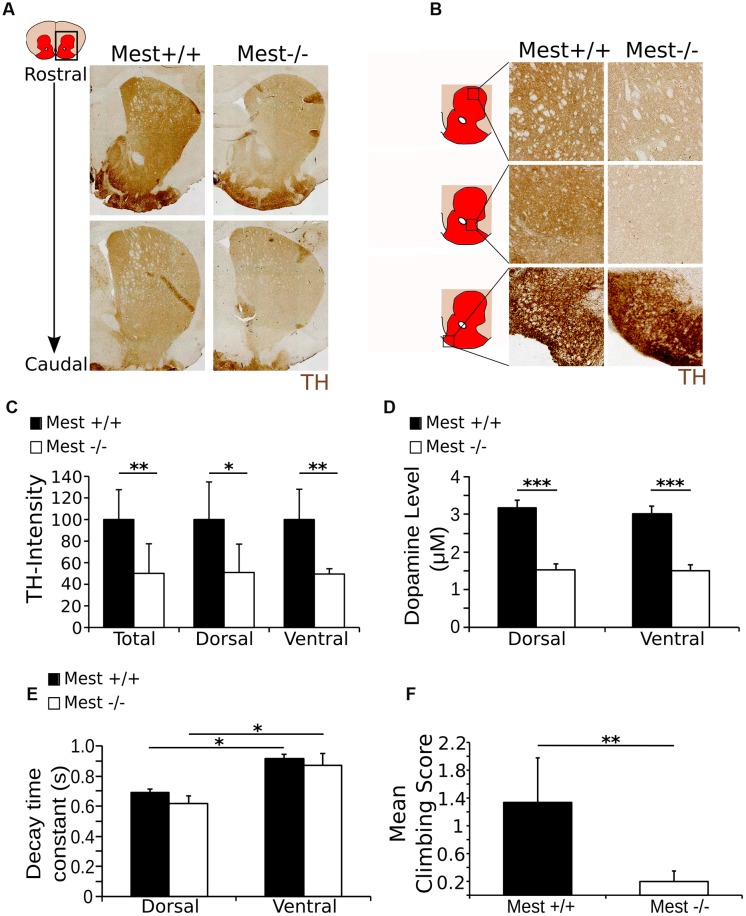
**The *Mest* KO has a 50% lower level of TH in the striatum than the WT control, reflected in the amount of DA release and climbing activity.** **(A)** TH protein (brown) in the striatum of the *Mest* KO compared to the WT control. **(B)** TH protein (brown) appears to be decreased throughout the striatum, indicating a general reduction of TH. However, TH presence in the tubercle of the striatum is not apparently altered. **(C)** Intensity measurements of TH protein level in the striatum of the *Mest* KO compared to the *Mest* WT show that the level of TH is significantly reduced in the total striatum with ∼50% (WT *n* = 4 black bars; KO *n* = 3 white bars; ^∗∗^*p* < 0.01), which is not specific for the dorsal (∼50%; ^∗^*p* < 0.05) or the ventral (∼49%; ^∗∗^*p* < 0.01) regions of the striatum. WT was set at 100%. **(D)** Cyclic voltammetry measurements in the striatum of the *Mest* KO (*n* = 4 white bars) and WT (*n* = 4 black bars) show that upon stimulation (500 μA) DA release is reduced with ∼50% in the *Mest* KO compared to WT controls (^∗∗∗^*p* < 0.001). WT animals have a DA release of ∼3 μM in both the dorsal and the ventral striatum, whereas *Mest* KO animals have a DA release of only ∼1.5 μM in both areas. **(E)** Time constant of the decay of the DA release in the dorsal and ventral striatum from Mest KO mice (*n* = 4 white bars) and WT controls (*n* = 4 black bars) (^∗^*p* > 0.05). Decay of DA release is not significantly altered between the WT and KO animals. Also, the characteristics of DA decay of the dorsal and ventral striatum are similar between the WT and KO. **(F)** Climbing test score. *Mest* KO (*n* = 4 white bar) adults show less (WT average score 1.3 compared to KO average score 0.2) activity in the climbing test than WT controls (*n* = 5 black bar) (^∗∗^*p* < 0.01).

At E14.5 the mdDA neuronal pool appears to be smaller and at E17.5 the SNc seems less cell-dense in the KO (**Supplementary Figure S1**). This initial effect of *Mest* deletion becomes more clear in adult mice (**Figure 2**). We examined the presence of mdDA neurons in 3- and 8-months old *Mest* KO and WT littermate controls. 3-months old *Mest* KO animals lose expression of *Th* specifically in the SNc (**Figure 2A** left panel, black arrowheads). 8-months old *Mest* KO animals show a more severe effect on *Th* than 3-months old *Mest* KO (**Figure 2A** right panel, black arrowheads), suggesting a progressive loss of *Th*. Similar defects were observed when *Dat* expression was analyzed in adjacent sections at these stages in *Mest* ablated animals (**Figure 2B**). Nissl staining on adjacent sections shows that in the *Mest* KO DA cell density is decreased (**Figure 2C-1** to **4**), indicating that loss of *Th* and *Dat* expression represents loss of mdDA neurons.

**FIGURE 5:**
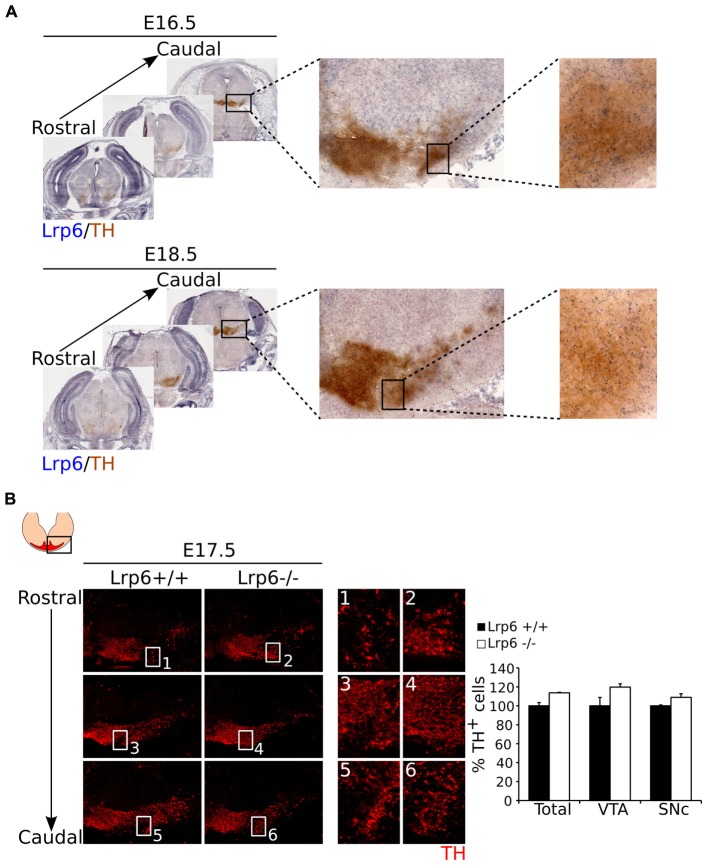
**Loss of *Lrp6* results in an increase of cell-density in the mdDA system at E17.5.** **(A)** Coronal view of *Lrp6* expression at late stages of development. *Lrp6* (blue) is ubiquitously expressed in the (mid)brain at E16.5 and E18.5 and shows overlap with the TH-expressing population (brown) of the future SNc, similar to what is seen for *Mest* expression. **(B)** Loss of *Lrp6* results in a modest increase in cell-density of TH-expressing neurons (red) in mdDA neuronal region at E17.5 **(1–6)**. Quantification of TH^+^ cells in the midbrain of E17.5 *Lrp6* KO (*n* = 2, white bars) and WT littermate controls (*n* = 2, black bars) shows an upward trend in the amount of mdDA neurons in the *Lrp6* KO compared to the WT. This effect is reflected in the TH-expressing cells in both the SNc and the VTA. WT was set at 100%.

To substantiate the mdDA cell-loss and to asses whether the defect is different between SNc and VTA, we performed immunohistochemistry for TH in 3- and 8-months old *Mest* KO and WT littermates (**Figure 3A**). TH-immunohistochemistry on 3- (**Figure 3A-1** to **4**) and 8-months (**Figure 3A-5** to **8**) old *Mest* KO animals shows a similar loss of TH-expressing cells as previously show on transcript level. Quantification of the amount of TH^+^ cells in the VTA and SNc showed that 3-months old *Mest* KO animals (*n* = 3) (**Figure 3B**) have significantly less (∼25% loss) TH-expressing cells than WT controls (*n* = 3; *p* < 0.01, one-tailed *t*-test). Separate quantification of the VTA and SNc (see Materials and Methods for definition), shows that loss of mdDA cells is significant for the SNc (∼32% loss *p* < 0.01, one-tailed *t*-test). Quantification of TH^+^ neurons in 8-months old *Mest* ablated animals (*n* = 3) (**Figure 3C**) shows a significant loss of TH-expressing cells throughout the mdDA neuronal population (∼32% loss *p* < 0.01, one-tailed *t*-test), which is most severe in the SNc (∼42% loss *p* < 0.01, one-tailed *t*-test), although we also detected a significant loss of ∼12% in the VTA at this stage (*p* < 0.01, one-tailed *t*-test). In comparison of the 3- and 8-months data the progressive loss of TH neurons is only detected in the SNc region (**Figure 3D**) (∼10% more loss in the 8-months SNc compared to the 3-months SNc *p* < 0.01, one-tailed *t*-test). Taken together, these data suggest that ablation of Mest causes a progressive loss of mdDA neurons in the SNc.

### The Loss of TH^+^ Neurons Is Reflected by Loss of DA Release in the Striatum

We showed that *Mest* ablation leads to a progressive loss of SNc DA neurons, which are known to project to the striatum in the adult brain (Ikemoto, 2007), suggesting that the dopaminergic output to this area may be affected in Mest KO animals. To confirm this possibility, we analyzed the presence of TH in the striatum in *Mest* KO animals (**Figures 4A,B**). In agreement with the above described cell loss, we find a 50% lower signal of TH protein in the striatum of *Mest* KO brains compared to controls (**Figure 4C**) (WT *n* = 3; KO *n* = 4; *p* < 0.01 one-tailed *t*-test). This effect was evenly distributed over the dorsal (50%; *p* < 0.05) and ventral (49%; *p* < 0.01) region (**Figure 4C**).

The observed lower TH protein levels in the striatum indicate that the DA output parameters may be affected in the striatum. To confirm this we analyzed the DA release parameters through cyclic voltammetry on dorsal and ventral regions of the striatum. In both regions WT animals (*n* = 4) show a DA-release of ∼3 μM after local field stimulation, whereas *Mest* KO animals (*n* = 4) display a decreased DA-release toward ∼1.5 μM (**Figure 4D**). This significant reduction in DA-release (50% less in the KO, *p* < 0.001 two-tailed *t*-test) confirms the functional consequence of the observed mdDA cell loss and observed lower TH protein levels in the striatum. Interestingly, although the DA release is impaired in *Mest* KO animals, the re-uptake machinery seems to be functionally intact, as the DA-decay is similar for both genotypes in both analyzed striatal areas (**Figure 4E**). General characteristics, like the peak amplitude of the oxidation current and the DA response (example of peak amplitude and DA response in the total and dorsal and ventral striatum in the WT: **Supplementary Figures S2A–C**) are equally unaltered in *Mest* KO animals (**Supplementary Figure S2D**). Taken together, the data show that the initial observed cell loss as a consequence of *Mest* ablation, is represented by a functional component of a reduction of DA-release in the striatum.

The observed loss of DA release in the striatum suggests that the animal would be affected in its behavior (Sonnier et al., 2007). In order to substantiate this, we investigated the climbing behavior in WT and *Mest* KO animals (Costall et al., 1978; Smidt et al., 2004). Adult *Mest* KO animals (*n* = 4) show a significantly lower climbing score after 30 min habituation than WT controls (*n* = 5) (*p* < 0.01, two-tailed *t*-test) (**Figure 4F**). The low climbing activity in the *Mest* KO mice indicates lower activation of DA receptors in the striatum, which is likely to be caused by the decrease in DA release as shown previously (Protais et al., 1976). Taken together, the reduced DA output is reflected by behavioral deficits in *Mest* ablated animals.

### *Lrp6* Ablated Animals Show Increased Cell-Density of TH^+^ Neurons

Thus far we have shown that *Mest* is involved in the development of the SNc, although the question remains how *Mest* is involved in this process. Studies performed by Jung et al. (2011) in adipocytes indicate that *Mest* regulates the balance of non-canonical and canonical WNT-signaling by inhibition of glycosylation and maturation of LRP6. If *Mest* has a similar function in mdDA neurons, loss of *Mest* would result in an increase of mature LRP6, thus favoring canonical over non-canonical WNT-signaling. Castelo-Branco et al. (2010) showed that loss of *Lrp6* leads to a delay in mdDA neuronal development and they claim that at E17.5 this effect was completely recovered. Based on the function of *Mest* during adipocytogenesis, we hypothesize that in developing mdDA neurons *Mest* has a similar function and loss of *Mest* may lead to an increase of LRP6 on the cell-membrane, eventually resulting in cell loss in the SNc (**Supplementary Figure S3**). If *Mest* indeed functions via regulation of LRP6, loss of *Lrp6* may lead to an increase of TH^+^ cells in the SNc, and thus an opposite phenotype compared to the *Mest* KO. To examine whether *Mest* functions (partially) via regulation of LRP6 maturation, we set out to determine whether *Lrp6* is expressed in similar areas as *Mest* at late developmental stages. To this end we analyzed the expression of *Lrp6* compared to the TH-expressing population at E16.5 and E18.5 (**Figure 5A**). *Lrp6* is ubiquitously expressed throughout the brain at both stages (**Figure 5A**). It is expressed in the midbrain at late developmental stages and overlaps with TH-expressing mdDA neurons at both E16.5 and E18.5.

Since *Lrp6* mutants are not viable (Castelo-Branco et al., 2010), we analyzed the mdDA neuronal population in the *Lrp6* KO at E17.5. Analysis of TH expression as a readout for mdDA neuronal presence (**Figure 5B**) shows that the mdDA neuronal pool appears to be broader and more cell-dense in *Lrp6* KO embryos at E17.5 compared to WT (**Figure 5B** blow-ups). The amount of TH^+^ neurons within the midbrain of *Lrp6* KO embryos (*n* = 2) shows a small upward trend compared to WT littermates (*n* = 2), which can be detected in both the VTA and the SNc (**Figure 5B** left panel). Taken together, these data show that *Lrp6* is expressed in the SNc during late stages of mdDA neuronal development and that loss of this gene possibly results in an increase in TH-expressing neurons at E17.5.

### *Mest* Is Essential of the Development of a SNc Subset Unaffected by Loss of *Pitx3*

**FIGURE 6:**
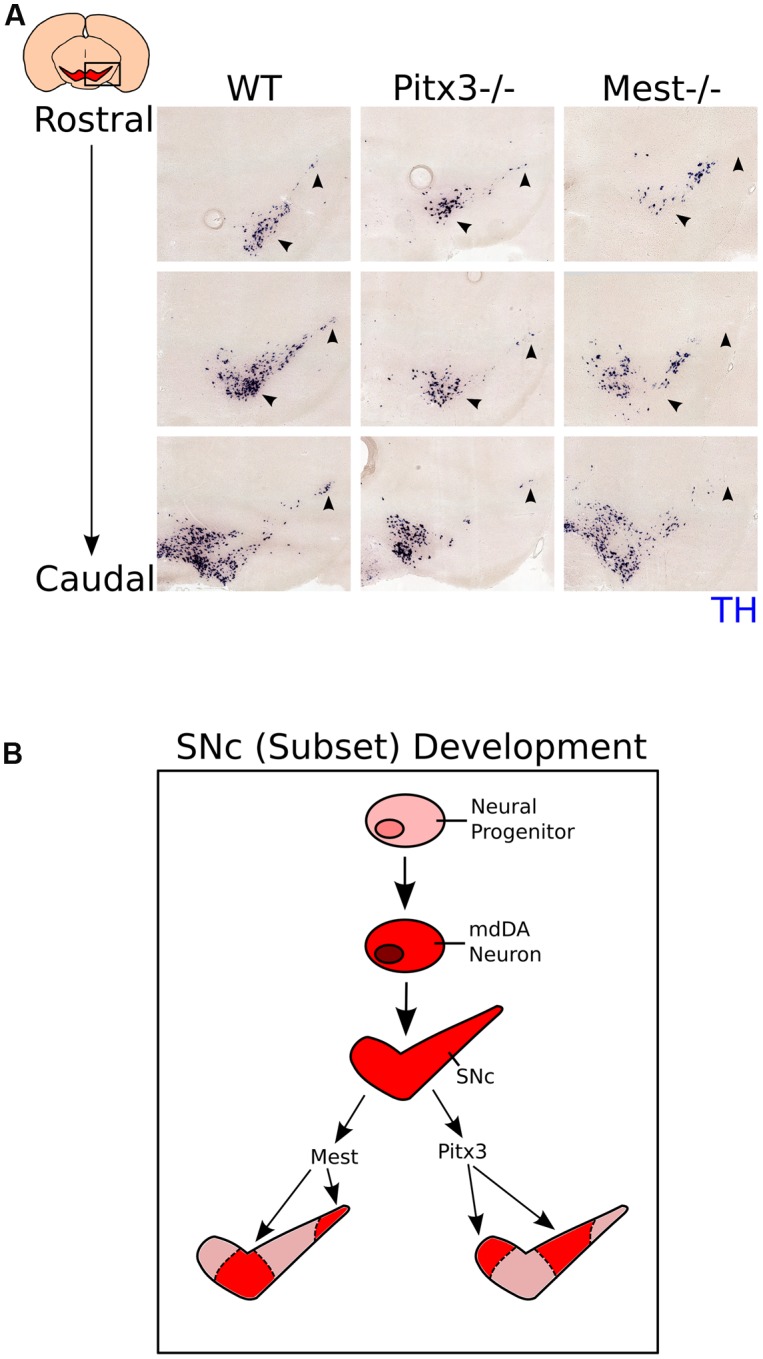
**Expression of *Th* in the *Pitx3* and *Mest* KO shows that different mdDA neuronal groups are affected in the two mutant mouse-lines.** **(A)** Expression of *Th* (blue) in the SNc of WT (3-months), *Pitx3* KO (Ak) (3-months), and *Mest* KO (8-months) animals. *Th*-expression is affected in different subsets of the SNc between the *Pitx3* and *Mest* KO animals (black arrowheads). **(B)** Schematic representation of subset development in the midbrain. Neural progenitors in the VZ of the midbrain start to specify into a mdDA neuron. Newly generated mdDA neurons will further specify into the VTA and SNc. The SNc consists out of at least two different subsets; one that is under the influence of *Pitx3*, and one under the influence of *Mest*.

Above we have shown that loss of *Mest* results in a progressive loss of mdDA neurons in parts of the SNc. Previously it has been shown that loss of *Pitx3*, a marker for mdDA neurons, similarly results in a specific loss of cells in the SNc, although this loss is not progressive (Smidt et al., 2004, Smits et al., 2005; Maxwell et al., 2005). In order to detect whether the neurons lost in the *Mest* mutant animals are part of the same population lost in the *Pitx3* mutant, we compared the SNc of *Pitx3* mutants at 3-months of age to the SNc of *Mest* mutants at 8-months of age (**Figure 6A**, anatomically matched). Interestingly, the region of *Th*-expressing neurons that is lost in the SNc of the *Mest* KO appears to be present in the *Pitx3* mutant (**Figure 6A** black arrowheads) and vice verse, suggesting that the SNc consists out of different neuronal subsets that are dependent on distinctive signaling for their maintenance (**Figure 6B**). Noteworthy, *Pitx3* is specifically expressed in neurons of the mdDA system (Smidt et al., 2004; Maxwell et al., 2005) and *Mest* is already expressed in the VZ of the midbrain and later becomes more restricted to SNc neurons. Taken together, these data indicate that the SNc consists out of different subsets that are specified by distinct molecular coding and have a specific vulnerability for neuronal degeneration (**Figure 6B**).

## Discussion

Mesodiencephalic dopaminergic (mdDA neurons are born from E10.5 onward from neural progenitors in the ventricular zone (VZ) of the embryonic midbrain (Bayer et al., 1995). During development these neurons make-up the different molecular subsets of the mdDA neuronal population, dependent on a unique set of signaling cascades and transcription factors (Smits et al., 2006; Di Salvio et al., 2010; Jacobs et al., 2011; Hoekstra et al., 2013; Smits et al., 2013; Veenvliet et al., 2013). The existence of these subsets is particularly apparent during Parkinson’s Disease (PD), in which neurons of the SNc show a specific vulnerability for neuronal degeneration, whereas neurons of the VTA remain relatively intact (Barzilai and Melamed, 2003). To understand the development of a neuronal progenitor toward an mdDA neuron which is part of a specific subset, our lab has previously conducted a genome-wide expression profiling study to elucidate possible factors that could be involved in the early specification and late differentiation into these subsets (Chakrabarty et al., 2012).

In the study presented here, we used these data to identify *Mest* as a factor involved in the development of the mdDA neuronal population. We have shown that *Mest* is expressed in the VZ of the embryonic midbrain at early developmental stages, whereas its expression becomes more restricted to the SNc at late developmental stages and in the adult stage. Examination of *Mest* KO embryos at E14.5 and E17.5 did not show any major effects on the mdDA neuronal population, indicating that if *Mest* has a function in early specification of neural progenitors to an mdDA neuron, this function is not crucial for their generation. Examination of mdDA neurons at adult stages (3-months and 8-months) shows that *Mest* mutants progressively lose TH-expressing neurons, which is specific for the SNc and not the VTA. These data indicate that *Mest* is involved in maintenance and survival of the nigral mdDA neurons. The loss of TH^+^ neurons in the SNc is accompanied by a decrease of ∼50% of TH protein present in the striatum and a decrease of ∼50% dopamine release, which is reflected by the lower activity of these animals in a climbing test (Protais et al., 1976). Taken together, these data suggest that *Mest* has a crucial role in the maintenance of SNc neurons. During the progression of PD, neurons in the ventral tier and medial tier of the SNc are lost before the disease affects the entire SNc (Braak et al., 2003, 2004; Vogt Weisenhorn et al., 2016). Interestingly, the progressive loss of mdDA neurons detected in the SNc of *Mest* neurons partly resembles this loss as seen in PD, indicating that the *Mest* ablated mouse model may serve to study degeneration events as observed in PD.

Since MEST is not a transcription factor, but a member of the α-β hydrolase protein family (Tilghman, 1999), it is unlikely that MEST activity will influence neuronal differentiation directly on the transcriptional level. However, it could function as a modulator of important signaling pathways during neuronal development and maintenance of the SNc. In adipocytes it has been shown that MEST modulates WNT-signaling by inhibition of glycosylation and maturation of LRP6, which inhibits canonical WNT-signaling and favors non-canonical WNT-signaling (Jung et al., 2011). Analysis of the *Lrp6* KO at E17.5 (Castelo-Branco et al., 2010) suggests that deletion of *Lrp6* results in a small increased cell-density in the mdDA neuronal population, indicating that loss of *Lrp6* results in a subtle opposite phenotype as observed in *Mest* ablated animals. These data might indicate that MEST influences WNT-signaling during mdDA neuronal development and maintenance. The importance of WNT modulation has been suggested in several reports about the differentiation of ESCs into DA neurons (Castelo-Branco et al., 2004; Schulte et al., 2005; Cajánek et al., 2009). Schulte et al. (2005) and Cajánek et al. (2009) claim that non-canonical WNT-signaling is necessary for the differentiation into DA cells, whereas Castelo-Branco et al. (2004) state that it is rather canonical WNT-signaling.

In this study we have established *Mest* as a novel factor in mdDA neurons, involved in the development and maintenance of a subset of the SNc. The progressive loss of TH-expressing neurons in this subset of the SNc and consequently the loss of DA release, makes the *Mest* KO a possible model to study disease progression, as observed in PD, and molecular determinants of defining selective vulnerability of nigral neurons. To further determine the role of *Mest* in the development of the SNc, future studies should focus on the mechanisms by which *Mest* regulates the differentiation of this specific subset of neurons. Furthermore, this gene could be examined in the human brain in relation to PD, in order to gain more insight whether *Mest* has a role in the development and/or progression of PD.

## Author Contributions

SM and MS designed, analyzed and interpreted the experiments and wrote the manuscript. JvH analyzed and interpreted the fast cyclic voltammetry data. All authors read and approved the final manuscript.

## Conflict of Interest Statement

The authors declare that the research was conducted in the absence of any commercial or financial relationships that could be construed as a potential conflict of interest.

## Supplementary Material

**FIGURE S1 | The effect of *Mest* on the mdDA system is relatively modest during development.** Coronal sections of E14.5 *Mest* KO and WT littermates show that, although the TH-expressing neuronal population (red) in the midbrain appears to be smaller (white arrowheads), there is no obvious change in expression of the DA marker TH. At E17.5 the effect on TH-expressing cells is small but apparent (white arrowheads), as cell-density of TH^+^ neurons appears to be decreased in the future SNc.

**FIGURE S2 | Example of the characteristics of the cyclic voltammograms measured in the DA response.** The DA response is normal in both the WT and KO striatum. **(A)** Representation of the cyclic voltammograms (recorded at 10 Hz) obtained after electrical stimulation of the striatum in WT animals. Numbers indicate the order of voltammograms after stimulation. **(B)** Peak amplitude of the oxidation current obtained from the voltammograms in **(A)**. **(C)** Example of the decay of DA response in the dorsal (white symbols) and ventral (black symbols) striatum in WT animals. Solid lines indicate a single exponential fit. **(D)** Peak amplitude of the DA response (as % of 500 μA) at different stimulation intensities in Mest KO mice (white symbols: *n* = 4) and WT littermates (black symbols: *n* = 4). Inset shows the same data on a logarithmic scale.

**FIGURE S3 | Schematic representation of WNT-signaling regulation in the presence and in the absence of MEST.** If MEST is present in the endoplasmic reticulum (ER) LRP6 glycosylation is inhibited. Therefore LRP6 maturation and migration to the cell membrane are prohibited, resulting in an inhibition of canonical WNT-signaling and increase of non-canonical WNT-signaling in DA neurons **(left)**. However, when MEST is not present in the ER, LRP6 can be glycosylated, mature and migrate to membrane. At the membrane it forms a complex with FZD, thereby inhibiting non-canonical WNT-signaling and favoring canonical WNT-signaling **(Right)**.
