# Supplementary figures and images for: Mest/Peg1 Is Essential for the Development and Maintenance of a SNc Neuronal Subset
Source: Front Mol Neurosci. 2017 Jan 13;9:166. doi: 10.3389/fnmol.2016.00166 (PMC5233686; doi:10.3389/fnmol.2016.00166)

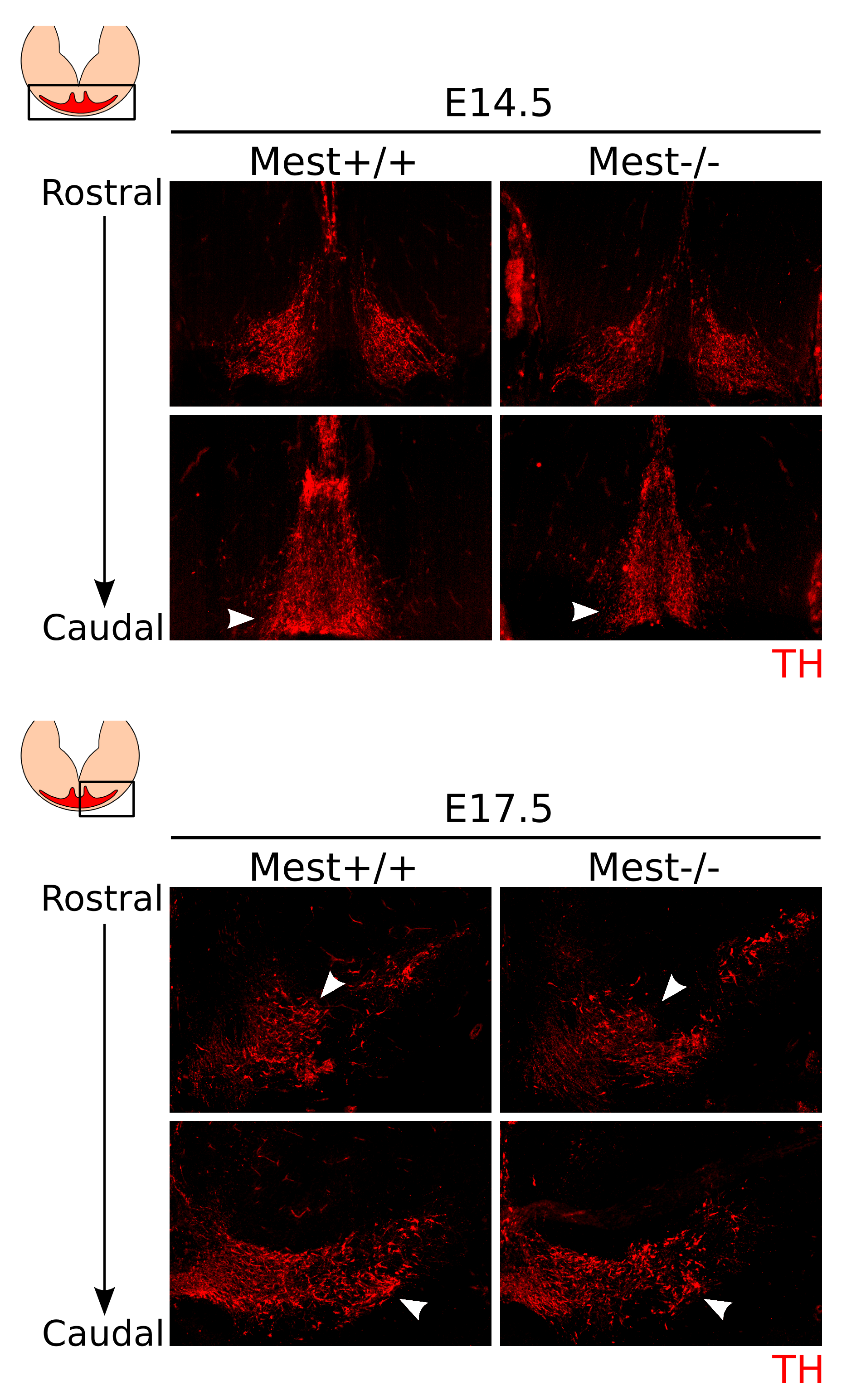

Supplement: Supplementary file 1 [file Image_1.TIF]

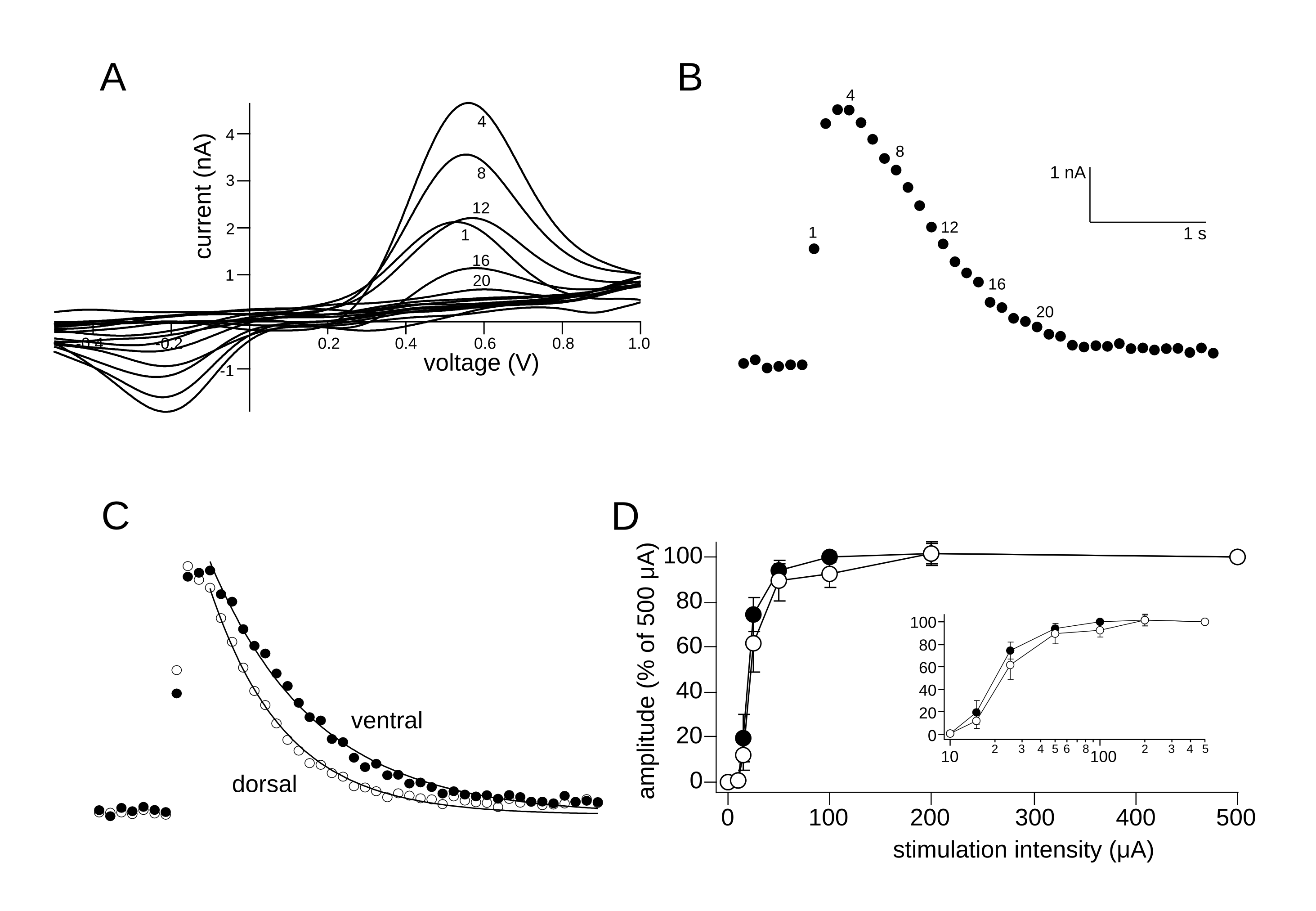

Supplement: Supplementary file 2 [file Image_2.TIF]

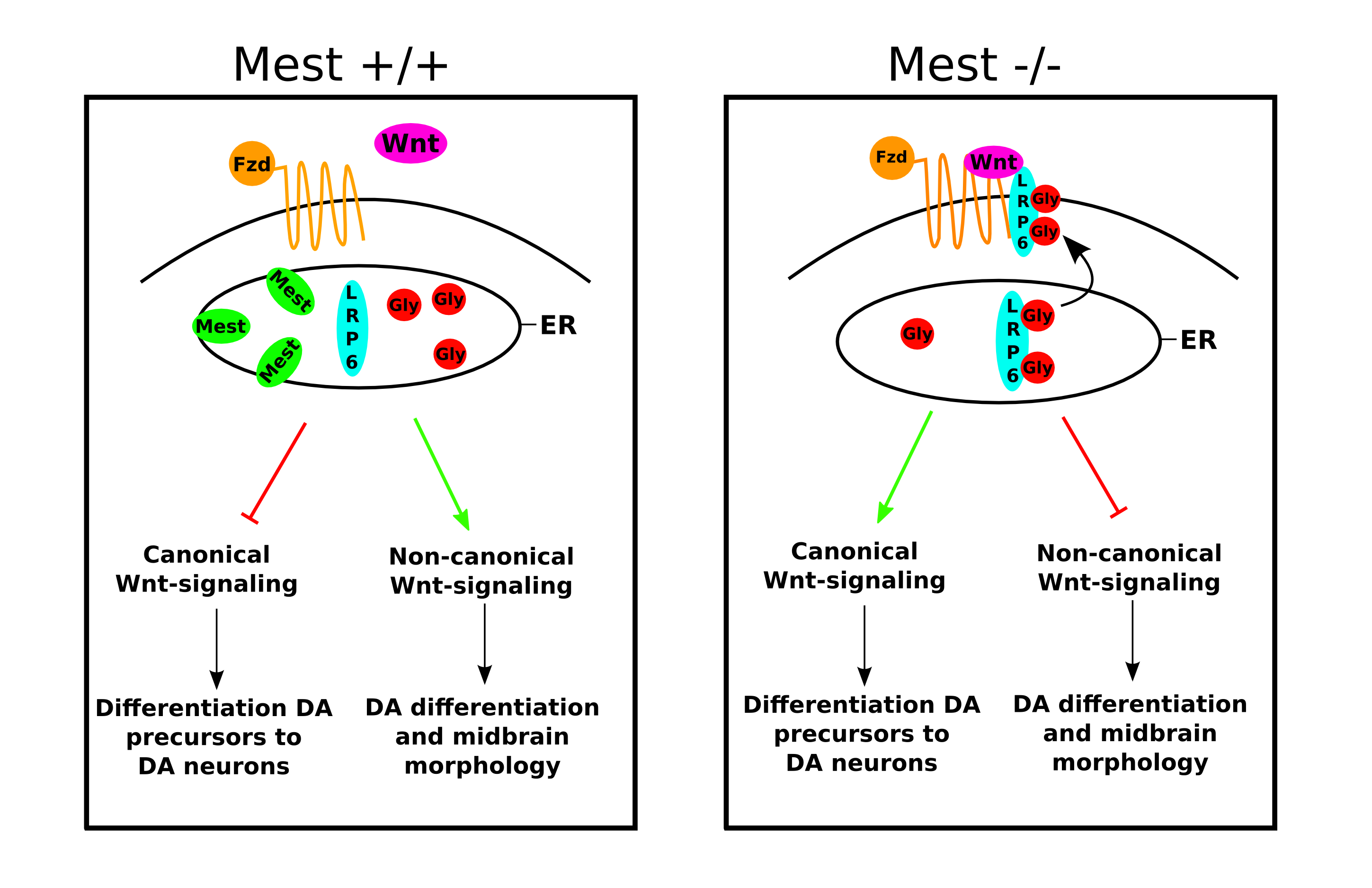

Supplement: Supplementary file 3 [file Image_3.TIF]
